# Carbonyl Stress and Microinflammation-Related Molecules as Potential Biomarkers in Schizophrenia

**DOI:** 10.3389/fpsyt.2018.00082

**Published:** 2018-03-13

**Authors:** Tohru Ohnuma, Shohei Nishimon, Mayu Takeda, Takahiro Sannohe, Narimasa Katsuta, Heii Arai

**Affiliations:** ^1^Juntendo University Schizophrenia Projects (JUSP), Department of Psychiatry, Faculty of Medicine, Juntendo University, Tokyo, Japan

**Keywords:** AGEs, biomarkers, carbonyl stress, glyceraldehyde-derived AGEs, microinflammation, pentosidine, schizophrenia, soluble tumor necrosis factor receptor 1

## Abstract

This literature review primarily aims to summarize our research, comprising both cross-sectional and longitudinal studies, and discuss the possibility of using microinflammation-related biomarkers as peripheral biomarkers in the diagnosis and monitoring of patients with schizophrenia. To date, several studies have been conducted on peripheral biomarkers to recognize the potential markers for the diagnosis of schizophrenia and to determine the state and effects of therapy in patients with schizophrenia. Research has established a correlation between carbonyl stress, an environmental factor, and the pathophysiology of neuropsychiatric diseases, including schizophrenia. In addition, studies on biomarkers related to these stresses have achieved results that are either replicable or exhibit consistent increases or decreases in patients with schizophrenia. For instance, pentosidine, an advanced glycation end product (AGE), is considerably elevated in patients with schizophrenia; however, low levels of vitamin B6 [a detoxifier of reactive carbonyl compounds (RCOs)] have also been reported in some patients with schizophrenia. Another study on peripheral markers of carbonyl stress in patients with schizophrenia revealed a correlation of higher levels of glyceraldehyde-derived AGEs with higher neurotoxicity and lower levels of soluble receptors capable of diminishing the effects of AGEs. Furthermore, studies on evoked microinflammation-related biomarkers (e.g., soluble tumor necrosis factor receptor 1) have reported relatively consistent results, suggesting the involvement of microinflammation in the pathophysiology of schizophrenia. We believe that our cross-sectional and longitudinal studies as well as various previous inflammation marker studies that could be interpreted from several perspectives, such as mild localized encephalitis and microvascular disturbance, highlighted the importance of early intervention as prevention and distinguished the possible exclusion of inflammations in schizophrenia.

## Introduction

Several studies have been performed to identify peripheral biomarkers for use in the diagnosis and monitoring of schizophrenia. These have mostly been based on pathophysiological hypotheses that schizophrenia is caused by disturbed neurotransmission, such as the dopaminergic (1) and glutamatergic (2–5) hypothesis, and thus have investigated the potential roles of peripheral monoamines and amino acids. Other studies have also investigated molecules related to the neurodevelopmental hypothesis, such as brain-derived neurotrophic factors (6, 7). Although some of these studies have shown altered biomarker levels in patients with schizophrenia, consistent results have not been achieved on replication, thereby raising questions over the validity of their use as diagnostic or therapeutic biomarkers (8–10). In addition, these studies have failed to show whether endogenous monoamine and/or amino acid levels in the peripheral blood truly reflect brain levels.

An alternative approach, based on the role of environmental factors, has also been proposed. Indeed, oxidative stress (11–13) and carbonyl stress (14–17), both environmental factors, have been associated with the pathophysiology of schizophrenia. Studies of biomarkers related to these stresses have achieved either results that are replicable or at least in the same direction (increases or decreases), concerning altered biomarker levels in schizophrenia. Moreover, relatively consistent results have been demonstrated in evoked microinflammation-related biomarker studies, with a subset of patients with schizophrenia showing pathophysiological microinflammation. In the present literature review, we discuss the findings of our previous studies, comprised of not only cross-sectional research, but also large-scale, longitudinal observations in which we identified putative biomarkers in the peripheral blood. We propose that these could be used for the diagnosis and monitoring of subpopulations of patients with schizophrenia.

## Carbonyl Stress

### Pentosidine and Pyridoxal

**Figure 1:**
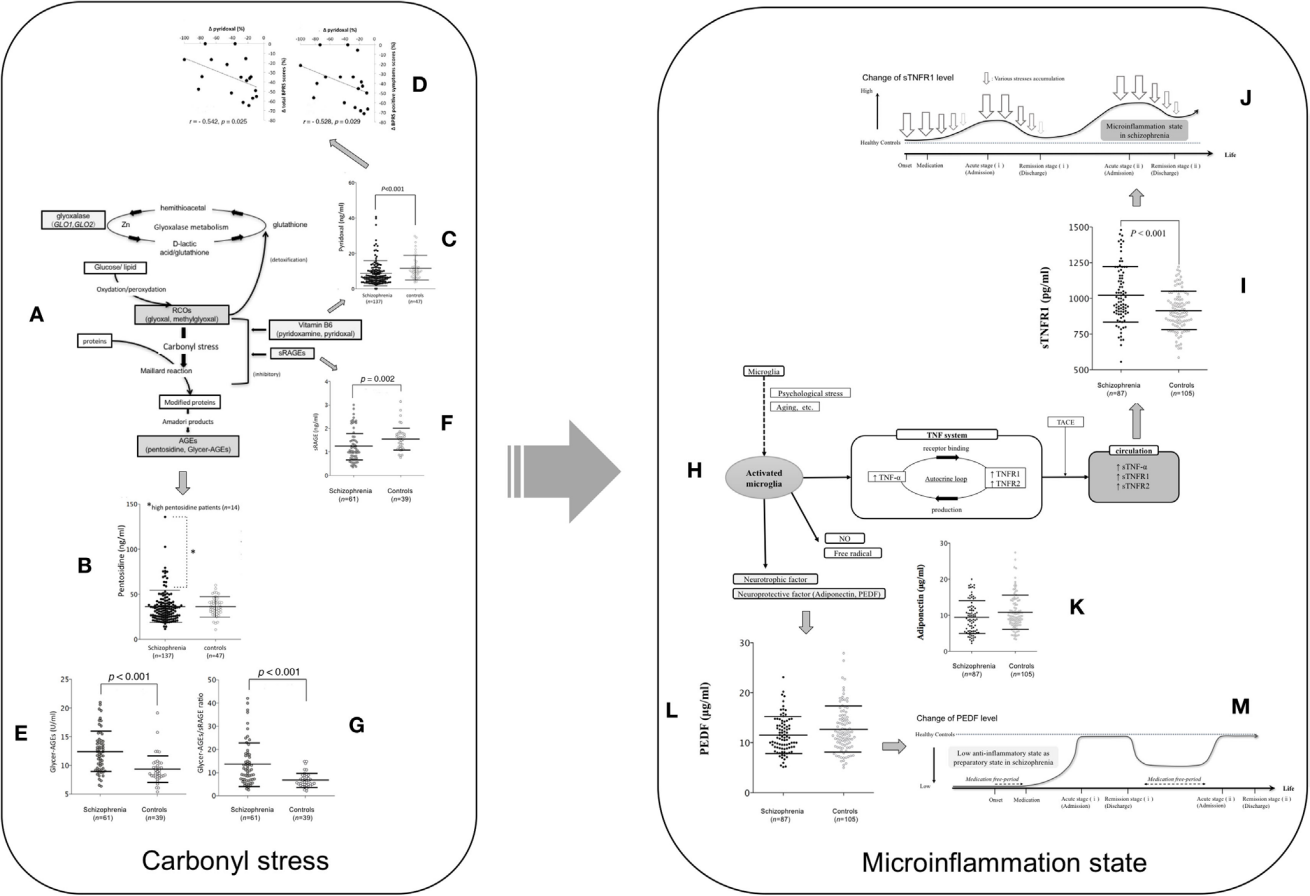
The assumed carbonyl stress and cytokine pathophysiology focused on TNF mechanism and their changes during the clinical course of patients with schizophrenia. **(A)** Carbonyl stress pathway. **(B)** Pentosidine between schizophrenia at admission and controls. Fourteen patients with high pentosidine levels (>2 SDs greater than the mean in controls) are indicated by an asterisk. Values were compared with the two-tailed Mann–Whitney *U* test. Error bars indicate mean and SDs (15). **(C)** Pyridoxal levels between schizophrenia at admission and controls. **(D)** Correlation between the changes (Δ) in pyridoxal levels and in total scores (left) and positive symptom scores (right) on the brief psychiatric rating scale among the paired patients who showed a decrease in pyridoxal levels (18 cases) according to the clinical course (15). **(E)** Glycer-AGE, **(F)** sRAGE, and **(G)** the Glycer-AGEs/sRAGE ratio are compared between patients with schizophrenia and controls (18). **(H)** Microinflammation pathway. **(I)** Soluble TNF receptor 1, **(K)** adiponectin, and **(L)** PEDF levels between schizophrenia at admission and controls (19). **(M)** Low anti-inflammatory state as preparatory state in schizophrenia. **(J)** Microinflammation process in schizophrenia (19). Abbreviations: RCOs, reactive carbonyl compounds; GLO1 and GLO2, glyoxalase enzymes, glyoxalase 1 and 2; Glycer-AGE, glyceraldehyde-derived AGE; sRAGE, soluble AGE receptors; TNF, tumor necrosis factor; NO, nitric oxide; TNF-α, tumor necrosis factor-α; TNFR1, tumor necrosis factor receptor 1; TNFR2, tumor necrosis factor receptor 2; PEDF, pigment epithelium-derived factor; TACE, tumor necrosis factor-α converting enzyme; sTNF-α, soluble tumor necrosis factor-α; sTNFR1, soluble TNF receptor 1; sTNFR2, soluble tumor necrosis factor receptor 2. Reprinted with some modifications from Ref. (15), Copyright (2014), with permission from Oxford University Press (license number; 4242240961739) (20), Copyright (2015), with permission from Elsevier (license number; 4241901371993), and (21), Copyright (2017), with permission from Elsevier (license number; 4241910166222).

Interesting results from a cross-sectional study showed that plasma levels of pentosidine, an advanced glycation end product (AGE), were significantly increased in patients with schizophrenia and that a subpopulation had low levels of vitamin B6 (14). In carbonyl stress pathway, reactive carbonyl compounds (RCOs), which cause carbonyl stress, are detoxified by degradation into lactic acid and glutathione by glyoxalase enzymes. Glyoxalase 1 and 2 (GLO1 and GLO2) are the rate-limiting enzymes in this metabolic pathway. Inhibition of RCO generation and the Maillard reaction by vitamin B6 results in the suppression of AGE accumulation (Figure 1A). This is important because vitamin B6 detoxifies RCOs. Among those with high pentosidine levels, most were also shown to have a family history of psychiatric illness, severe symptoms, and an affected gene associated with RCOs (14). A subsequent cross-sectional study that included more clinical data and more patients with chronic schizophrenia showed that the presence of carbonyl stress could lead to treatment resistance, establishing a role for markers carbonyl stress in chronic schizophrenia (16). These studies evidenced that high pentosidine and low pyridoxal levels in the peripheral blood could be state markers of “treatment resistance” in some patients with schizophrenia. However, to verify whether altered pentosidine and pyridoxal levels could be “state” and/or “therapeutic” biological markers of schizophrenia, parallel cross-sectional and longitudinal studies were needed that followed patients with schizophrenia from acute illness to remission.

#### Pentosidine

We repeated our original cross-sectional study to investigate the means and clinical significance of serum markers of carbonyl stress in 137 patients with acute schizophrenia and in 47 healthy controls (15). Although serum pentosidine levels were markedly elevated in some patients, levels were not significantly altered in schizophrenia (Figure 1B), meaning that pentosidine could not be confirmed as a state biomarker for severity. Moreover, the putative marker showed no correlation with any other clinical feature of schizophrenia (e.g., age at onset, illness duration, and family history). There was, however, a significant positive correlation between pentosidine levels and both the daily antipsychotic dose and the cumulative antipsychotic exposure (duration multiplied by the daily dose) (15).

The positive correlation between pentosidine levels and daily antipsychotic dose has been reported in previous studies (14–17), which concluded that this was due to the characteristic that treatment-resistant patients are usually treated with relatively high daily antipsychotic doses. To test this hypothesis, the influence of first- and second-generation antipsychotics, as well as the influence of different combinations of medication and dosages, was analyzed in a follow up to our first cross-sectional study (15). We enrolled another 137 patients with acute schizophrenia and pooled them with the existing cohort (22). We evaluated the associations of serum pentosidine with clinical variables that have shown a significant association with peripheral pentosidine levels (14–17). These included the severity of symptoms, the duration of education, and the duration and daily doses of antipsychotic, antiparkinsonian, and anxiolytic medications. The pooled cohort (*n* = 274) showed associations between higher serum pentosidine levels and both higher daily antipsychotic doses and longer estimated durations of medication use, only in the context of antipsychotic polypharmacy (not with monotherapy). However, there was no statistical significance for diagnostic purposes (Table 1) (22).

**Table 1:** Multiple linear regression analysis of possible explanatory variables for the serum pentosidine levels.

	Total		Poly	
	(*N* = 274)		(*N* = 68)	
	
Independent variables:	*b*(SE)	β	*b*(SE)	β
Duration of education	–	–	–	–
Total BPRS score	–	–	–	–
Estimated duration of medication	0.318 (0.139)	0.197*	0.900 (0.443)	0.365*
Daily dose of antipsychotics	0.012 (0.003)	0.390***	0.014 (0.007)	0.368*
Daily dose of antiparkinsonian drugs	–	–	–	–
Daily dose of anxiolytics	–	–	–	–

Constant	32.0		16.2	
*R*^2^	0.195***		0.407***	

Multiple linear regression analysis includes six possible explanatory variables for all 274 patients and for the polypharmacy treatment group. Statistical values of independent factors excluded from the first step of the equation are not shown. *p < 0.05, **p < 0.01, ***p < 0.001 (22).

*BPRS, brief psychiatric rating scale; Poly, polypharmacy treatment group (treated with both types of antipsychotics; at least one of each); SE; standard error. Reprinted from Ref. (20), Copyright (2017), with permission from Elsevier (license number; 4241900807314)*.

#### Pyridoxal

As mentioned, our first cross-sectional study also showed a potential role for low levels of pyridoxal, a form of vitamin B6 (15). In the first replication study (Figure 1C) (15) with a pooled cohort of 274 patients (22), the results were consistent, showing a significant decrease in pyridoxal levels among patients with schizophrenia. Although no study had shown a correlation with other clinical features of the disease (14–17, 22) in our longitudinal study, we managed to show that the low pyridoxal levels during acute schizophrenia increased according to the clinical course of the illness but were not directly correlated with symptom improvement. Moreover, 18 patients whose pyridoxal levels decreased during their illnesses had less symptom improvement (Figure 1D) (15). Thus, we concluded that decreasing pyridoxal levels during the clinical course of schizophrenia could be a biomarker for non-responders to antipsychotic therapy.

### Glyceraldehyde-Derived AGEs (Glycer-AGEs)

Recently, strong *in vivo* neurotoxicity has been shown with Glycer-AGEs (23) that are central to the pathophysiology of neurodegenerative diseases (18, 24). In a longitudinal cross-sectional study of peripheral serum Glycer-AGE levels, we included 61 patients with acute schizophrenia and 39 controls, with follow up data for 54 patients to remission (20). Peripheral Glycer-AGE levels were significantly higher than those of controls (Figure 1E), but did not change with the clinical disease course and were not correlated with clinical features, indicating that they may not be useful as therapeutic or state markers in patients with schizophrenia (20); however, they could serve as diagnostic markers. Indeed, unlike pentosidine, their levels were not correlated with daily chlorpromazine doses, indicating the lack of an iatrogenic effect (15).

### Soluble Receptors for AGE Receptors

AGEs interact with AGE receptors (RAGEs) to increase oxidative and carbonyl stress (25). Circulating receptors are also bound, such as endogenous secretory RAGE (esRAGE) and soluble receptors for RAGE (sRAGE), with the latter most likely to be indicative of carbonyl stress because they exist at levels five times greater than those of esRAGE (25). Peripheral serum soluble AGE receptors (sRAGE) levels were investigated in the earlier study of Glycer-AGEs (20), where levels in patients with acute schizophrenia were found to be significantly lower than those in healthy controls (Figure 1F). Accordingly, significant negative correlations were identified between serum Glycer-AGE and sRAGE levels, with Glycer-AGEs/sRAGE ratios being sensitive markers of carbonyl stress. Indeed, the ratio differed significantly, with an approximately twofold higher ratio in patients with schizophrenia than in healthy controls (Figure 1G). Neither sRAGE levels nor Glycer-AGEs/sRAGE ratios showed any correlation with clinical symptoms or change with the clinical course, meaning that they could not be used as state makers of schizophrenia.

Interestingly, discriminant analyses confirmed that Glycer-AGEs and Glycer-AGEs/sRAGE ratios were significant diagnostic markers for schizophrenia, effectively distinguishing between patients and controls in 70% of cases. Thus, Glycer-AGEs and their ratio to sRAGE could be used as diagnostic markers of schizophrenia.

## Microinflammation

The oxidative (11, 26) and carbonyl stresses (15, 20, 22) involved in the pathophysiology of schizophrenia have been considered to induce a proinflammatory state that could lead to microinflammation (Figure 1H). In microinflammation pathway, when the brain is exposed to psychological stress or aging, the microglia develop to an activated state. The activated microglia release various inflammatory cytokines, nitric oxide, free radicals, neurotrophic factor, and neuroprotective factor. Tumor necrosis factor-alpha (TNF-α) binds to TNFR1 and TNFR2 and the TNFRs induce TNF-α production. These “autocrine loops” chronically continue to act on the TNF system. TNF-α, TNFR1, and TNFR2 are cleaved by TNF-α converting enzyme, and subsequently exist as soluble tumor necrosis factor-α, soluble TNF receptor 1 (sTNFR1), and sTNF2 in circulation. These systems ultimately induce neuroinflammation. Several investigations of inflammatory markers in the peripheral blood have assessed the chronic inflammatory statuses of patients with schizophrenia (27–30). In a recent review and meta-analysis, interleukin (IL)-1β, IL-6, and transforming growth factor-β were identified as putative state markers, whereas IL-12, interferon-γ, tumor necrosis factor (TNF)-α, and soluble IL-2 receptors were identified as putative trait markers based on evidence from longitudinal observations of patients with acute schizophrenia (31). However, whether changes in these biomarker levels reflect the current pathological disease state has not been established when controlled for age, sex, and body mass index (BMI). It is, therefore, unclear if these can be useful as biological markers for acute schizophrenia in clinical practice. In our recent longitudinal cross-sectional study of microinflammatory biomarkers, we investigated whether serum levels of sTNFR1, adiponectin, and pigment epithelium-derived factor (PEDF) could be used as diagnostic and/or prognostic biomarkers for acute schizophrenia.

### Soluble TNF Receptor 1

Serum TNF-α has been shown to have proinflammatory effects. These effects occur when it binds to TNF receptors 1 or 2 (32), which enter the circulation as soluble TNFRs (sTNFRs) (33). Given that the most stable and reliable marker of TNF-α activity is considered to be sTNFR1 (34, 35), biomarker studies were done to investigate the average levels (means) of neuroinflammation in patients with schizophrenia (19, 34–36), concluding that sTNFR1 could reflect treatment resistance or severe clinical disease trajectories (19, 35).

In cross-sectional research, we reported significant differences in peripheral sTNFR1 levels between patients with acute schizophrenia and controls, regardless of whether we adjusted for physical confounders that could affect microinflammation (e.g., BMI and age) (21). Peripheral sTNFR1 levels were significantly higher in the patients with acute schizophrenia than in healthy controls (Figure 1I), but high sTNFR1 alone showed moderate discriminating efficacy between the two groups (>60% accuracy). The marker also effectively discriminated healthy controls from patients with clinical deterioration (>90% accuracy) and from patients with clinical improvement (>80% accuracy) during inpatient care. Thus, higher serum sTNFR1 levels could predict so-called treatment-resistant schizophrenia, as supported by the results of previous studies (19, 35). Although the peripheral sTNFR1 levels did not reflect clinical severity in acute schizophrenia, levels were correlated with the duration of illness and age, consistent with previous research showing an association between inflammatory biomarkers and age in patients with psychiatric disease (27). However, healthy controls showed no such association between age and peripheral sTNFR1 levels, and patients who were not taking medication had significantly shorter illness durations and lower sTNFR1 levels compared with medicated patients (21). Thus, we concluded that sTNFR1 levels may only increase as the disease progresses and stressors accumulate (Figure 1J) (21).

In longitudinal research, we found that intensive antipsychotic therapy between admission and discharge produced significant decreases in elevated peripheral sTNFR1 levels (21). Unfortunately, sTNFR1 did not reflect symptomatic improvement, with changes in levels failing to correlate with changes in positive, negative, or total scores on the Brief Psychiatric Rating Scale, scores on the Global Assessment of Functioning scale, or with daily antipsychotic doses. We, therefore, concluded that overall relief of psychological stress factors was reflected by changes in peripheral sTNFR1 levels, with abating symptoms being only one such factor. Importantly, hospitalization did not appear to be a cause of stress (Figure 1J).

### Adiponectin

Adiponectin has been shown to regulate insulin sensitivity and tissue inflammation (37), but in our studies, we found no significant differences in adiponectin levels, irrespective of whether it was matched for potential confounders (Figure 1K). However, there was a significant correlation between BMI and serum adiponectin levels independent of the disease (21). Several studies have indicated that peripheral adiponectin levels are elevated in patients with chronic schizophrenia, concluding that adiponectin could be a marker for the metabolic syndrome for schizophrenia, especially that triggered by atypical antipsychotics, rather than reflecting the disease (38–42).

### Pigment Epithelium-Derived Factor

Pigment epithelium-derived factor inhibits the AGE–RAGE pathway in the activation of proinflammatory genes, with higher peripheral AGE/sRAGE ratios reported in patients with schizophrenia (20). By suppressing proinflammatory pathways, such as those involved in carbonyl stress, PEDF may be able to inhibit inflammation (43), and may function as an anti-inflammatory in acute schizophrenia. In the above-mentioned study of sTNFR1 (21), peripheral PEDF levels were not significantly different between patients with acute schizophrenia and controls, regardless of matching for confounders (e.g., BMI and age; Figure 1L). Interestingly, peripheral PEDF levels in the 42 patients not receiving medication were significantly lower compared with controls, but without producing higher sTNFR1 levels (21). It is possible that the lower PEDF levels in untreated patients with shorter disease durations reflect a preparatory state for an inflammatory pathophysiology, from which prolonged and severe disease-related stress causes chronic inflammation reflected by increased peripheral sTNFR1 in patients receiving antipsychotics (Figure 1M). In addition, the fact that PEDF did not alter over time and did not correlate with other clinical variables may indicate that lower levels only affect disease onset (Figure 1M) (21). Taken together mentioned-above cross-sectional and longitudinal changes in sTNFR1 and PEDF levels in schizophrenia, we hypothesized that; while all patients with schizophrenia were initially treated with medication on admission, it was noted that patients not taking medications, including drug-naïve patients and those with relapse, had low PEDF levels (Figure 1M), but similar sTNFR1 levels (Figure 1J) when compared with healthy controls. This indicated a low anti-inflammatory state among these patients. However, during the acute exacerbation, greater stress levels (Figure 1J; arrows) were associated with higher sTNFR1 levels compared to healthy controls. Subsequently, while sTNFR1 and psychological stress levels decreased by the time of remission, PEDF levels did not change despite intensive antipsychotic therapy (Figure 1M).

### Involvement of Carbonyl Stress and Microinflammation in Schizophrenia: Model of Mild Localized Encephalitis With Microvascular Damage

Several epidemiological studies have suggested the involvement of prenatal (*in utero*) infection-related inflammation in the pathophysiology of schizophrenia (44, 45). In particular, a study highlighted the prenatal exposure to herpes simplex virus type 1 infection as one of the etiologies of onset (46). Clinically, patients with mild localized encephalitis, especially localized temporal lobe encephalitis caused by herpes simplex virus type 1, have been shown to exhibit schizophrenic-like symptoms, such as auditory hallucinations and cognitive impairments (47–49). Environmental factors, such as physical stress (including these infections), hypoxia, and oxidative as well as carbonyl stress are known to evoke microinflammation that can damage the neurons and microvascular systems, with the latter damage easily (50–52). Reportedly, these findings could corroborate the already hypothesized “vascular-inflammatory theory” in the central nervous system of patients with schizophrenia (53). Despite the relatively mild degree of inflammation caused by each environmental factor, the cumulative microinflammation due to the repeated exposure to various postnatal stresses could evoke the exacerbation and/or treatment resistance in patients with schizophrenia (Figure 1J). Overall, while the damage to temporal lobe microvascular system might be only partly involved in the pathophysiology of onset of schizophrenia, the prevention of exposure to these microinflammations could be essential in patients with schizophrenia.

### Microinflammations as Specific Pathophysiology in Schizophrenia

Reportedly, environmental stress factors and evoked microinflammations could be involved in several psychiatric disorders, such as depression (54) and bipolar disorders, and some of these disorders exhibit characteristics similar to those exhibited in schizophrenia with altered cytokines (36). For instance, not only apparent neurodegenerative diseases, but also other psychiatric disorders could involve altered cytokine systems as their pathophysiology. In fact, patients with post-traumatic stress disorder, typically caused only due to intense psychological stress, also exhibited an elevation in peripheral TNF-α levels, which were reproducibly reported in other psychiatric diseases as well, including schizophrenia (36, 55). Thus, further studies are warranted to investigate the difference in the degree of alteration of cytokine levels among various neuropsychiatric disorders. Perhaps, findings of future research could be a diagnostic marker in the clinical practice if the discriminant analysis established a significant difference between several subjects and same measurement methods.

### Immune Responses Affected by Microinflammation in Schizophrenia

Although the immune responses directly affected by aforementioned stresses cannot be inferred, an interesting epidemiological study established an association of atopic disorders (in general) and asthma (in particular), namely type 1 hypersensitivity, with the risk of developing schizophrenia (56). In addition, elevated type-2 cytokine levels in schizophrenia were also reported to be a part of the pathophysiology (57). While psychosocial stress alone also could lead to asthma exacerbation by accompanying with histological microvascular system inflammation and increase of serum type-2 cytokine levels in asthma model animal (58). Taken together these findings, the damaged microvascular system seems to be the crucial site of inflammation in asthma and in the schizophrenia, especially in exacerbation.

Based on our previous studies and other above-mentioned studies on psychiatric subjects, increased TNF superfamily levels, among several cytokines, could be, at least, involved in the pathophysiology of psychiatric diseases (Figure 1H).

### Early Prediction and Prevention of Microinflammation

Among markers of microinflammation, the most reproducible outcomes have been obtained for elevated serum sTNFR1 levels as a marker of treatment resistance in patients with schizophrenia (21, 34, 36). Apparently, early intervention for diseases associated with carbonyl stress and microinflammation, such as diabetes mellitus, is imperative to prevent irreversible complications. Consequently, earlier discovery of proinflammatory states with sensitive markers of carbonyl stress, before any disease reaches the inflammatory stage, could be clinically beneficial. Reportedly, regarding the carbonyl stress status, Glycer-AGEs exert strong neurotoxicity (23), and Glycer-AGEs as well as the Glycer-AGE:sRAGE ratio demonstrate higher sensitivities than other AGEs-related molecules in schizophrenia (20). Thus, serum Glycer-AGEs and the Glycer-AGE:sRAGE ratio could act as important proinflammatory markers of subsequent microinflammation that is potentially associated with treatment-resistant schizophrenia. In addition, some studies have revealed that early intervention for prevention of exposure to these stresses, e.g., diet and habits, as well as a higher exposure to the daily dose of antipsychotics (22, 59) would be beneficial for such patients. Recently, an interesting study reported that augmentation therapy with high-dose pyridoxamine (a form of vitamin B6 that detoxifies RCOs) could improve, in part, patients with high AGEs levels and be a novel strategy for treatment-resistant schizophrenia (60). Furthermore, a study reporting that for patients in a state of micronflammation, add-on-therapy with Cox-II-blockers (nonsteroidal anti-inflammatory drugs) or valacyclovir (an antiviral drug), which improved acute schizophrenia, could be used as a treatment strategy based on the above-mentioned mild localized encephalitis model (61).

## Conclusion

In this review, we primarily offered a summary and analysis of our previous research into markers of proinflammatory carbonyl stress and microinflammation, and now highlight six key conclusions. First, in patients receiving antipsychotic polypharmacy, high peripheral pentosidine levels may be associated with high daily doses and longer antipsychotic use. Second, decreasing pyridoxal levels during treatment could potentially identify non-responders to antipsychotic therapy. Third, although apparently unsuitable for use as markers of proinflammatory states, higher Glycer-AGE levels and higher Glycer-AGEs/sRAGE ratios could be diagnostic of schizophrenia. Fourth, as previously reported (19, 35), elevated sTNFR1 levels are accurate at discriminating patients who deteriorate during inpatient care from both healthy controls and patients who improve. Thus, higher peripheral sTNFR1 levels may not only be a useful adjunctive diagnostic biomarker for acute schizophrenia, but may also be a valuable prognostic biomarker for treatment response. Fifth, low PEDF levels in untreated patients with short disease durations might reflect a preparatory state for inflammation. Finally, raised adiponectin levels could be useful as a marker of the metabolic syndrome in patients receiving antipsychotics for schizophrenia. Our cross-sectional and longitudinal studies as well as various previous inflammation marker studies that could be interpreted from several perspectives, such as mild localized encephalitis and microvascular disturbance, highlighted the importance of early intervention as prevention and distinguished the possible exclusion of inflammations in schizophrenia.

## Author Contributions

TO contributed to the interpretation of the data and writing of the paper. SN, MT, TS, and NK contributed to the clinical evaluation of patients and the conception of the study. HA contributed to the conception and design of the study. All authors contributed to and approved the final manuscript.

## Conflict of Interest Statement

None of the authors have any conflicts of interest pertaining to this paper to disclose.
